# Physician Visits and Colorectal Cancer Testing Among Medicare Enrollees in North Carolina and South Carolina, 2005

**Published:** 2011-08-15

**Authors:** Anna P. Schenck, Carrie N. Klabunde, Joan L. Warren, Eric Jackson, Sharon Peacock, Pauline Lapin

**Affiliations:** University of North Carolina, Gillings School of Global Public Health. Dr Schenck is also affiliated with the Carolinas Center for Medical Excellence, Cary, North Carolina; National Cancer Institute, Bethesda, Maryland; National Cancer Institute, Bethesda, Maryland; the Carolinas Center for Medical Excellence, Cary, North Carolina; the Carolinas Center for Medical Excellence, Cary, North Carolina; Centers for Medicare and Medicaid Services, Baltimore, Maryland

## Abstract

**Introduction:**

Many Medicare enrollees do not receive colorectal cancer tests at recommended intervals despite having Medicare screening coverage. Little is known about the physician visits of Medicare enrollees who are untested. Our study objective was to evaluate physician visits of enrollees who lack appropriate testing to identify opportunities to increase colorectal cancer testing.

**Methods:**

We used North Carolina and South Carolina Medicare data to compare type and frequency of physician visits for Medicare enrollees with and without a colorectal cancer test in 2005. Type of physician visit was defined by the physician specialty as primary care, mixed specialty (more than 1 specialty, 1 of which was primary care), and nonprimary care. We used multivariate modeling to assess the influence of type and frequency of physician visits on colorectal cancer testing.

**Results:**

Approximately half (46.5%) of enrollees lacked appropriate colorectal cancer testing. Among the untested group, 19.8% had no physician visits in 2005. Enrollees with primary care visits were more likely to be tested than those without a primary care visit. Many enrollees who had primary care visits remained untested. Enrollees with visits to all physician types had a greater likelihood of having colorectal cancer testing.

**Conclusions:**

We identified 3 categories of Medicare enrollees without appropriate colorectal cancer testing: those with no visits, those who see primary care physicians only, and those with multiple visits to physicians with primary and nonprimary care specialties. Different strategies are needed for each category to increase colorectal cancer testing in the Medicare population.

## Introduction

Costs associated with colorectal cancer (CRC) are projected to be between $11.4 and $14 billion in 2020, creating a substantial burden for Medicare enrollees and the Medicare program (1). Illness and death associated with CRC can be reduced through early detection and treatment. Four recommended tests for CRC screening of persons of average risk (2) are covered under Medicare: 1) annual fecal occult blood testing (FOBT); 2) sigmoidoscopy or 3) barium enema every 4 years; or 4) colonoscopy every 10 years. Despite having Medicare coverage for CRC tests, less than half of Medicare enrollees have received CRC tests at the recommended intervals (3)

Much debate about health care reform centered on providing health insurance coverage to uninsured Americans. Although lack of insurance coverage is a leading reason for lack of access to health care, insurance coverage does not ensure access to health care. Aday et al (4) have used the term "realized access" to highlight the importance of factors in addition to insurance coverage and availability of providers that influence consumer access to health care, including the "transactions between patients and providers during the process of care delivery."

Physician influence on patients' CRC screening status is well documented. Physician recommendation has been associated with increased likelihood of screening (5,6), and the lack of recommendation has been identified as a barrier (7,8). Having a recent well visit (6), being seen in group practices rather than solo practices (9), and visiting internal medicine physicians rather than other primary care providers (10) have all been linked with increased CRC testing. Understanding physicians' influence on the CRC screening status of Medicare enrollees is, however, complicated by the fragmented care received by most Medicare enrollees. One study showed that most Medicare enrollees see 7 physicians in 4 practices in a given year (9).

The objective of this study was to examine the role of physician visits among Medicare enrollees with and without CRC testing — enrollees who access this preventive service and enrollees with "unrealized access." Our aim was to identify potential opportunities to increase CRC testing in the untested population.

## Methods

### Study population

In this study, we included North Carolina and South Carolina Medicare enrollees aged 65 to 85 as of January 1, 2005. We limited analyses to enrollees with at least 11 months of Part B Medicare coverage during calendar years 2004 and 2005, and no health management organization (HMO) enrollment. We excluded people with a diagnosis of CRC on any Medicare claim in the year before their CRC test or in 2004 for enrollees with no test (n = 5,614), people with selected medical conditions (Appendix A) considered to confer higher risk for CRC (n = 4,734), and people with more than 100 physician visits in the year of observation (n = 20). This study was completed at The Carolinas Center for Medical Excellence under the Quality Improvement Contract for the States of North and South Carolina and therefore needed no institutional review board approval.

### Data sources

From Medicare's Enrollment Database, we obtained demographic information, reason for entitlement (age or disability), Part B and HMO enrollment, state of residence, and eligibility for state buy-in (Medicaid). To identify health services, we used a summary file available to quality improvement organizations containing claims for all services from physician offices, clinics, and hospitals and a unique physician identification number (UPIN). We used the disease and procedure codes on 2004 inpatient Medicare claims and evaluation and management outpatient Medicare claims to create a comorbidity index for the study group, following a method developed by Klabunde and colleagues (11). We calculated a comorbidity score for each enrollee by summing the count of the disease flags, weighting clinical conditions equally. We classified enrollees without eligible claims as having unknown comorbidity status.

### Classification of enrollee CRC test use

To identify CRC tests (FOBT, sigmoidoscopy, colonoscopy, and barium enema) in 2005, we used Medicare claims from all clinical settings according to procedure codes (Appendix B) and dates of service listed on the claims. We included diagnostic and screening procedures because the reason for the test cannot be reliably determined from Medicare claims (12,13). To ascertain CRC testing before 2005, we reviewed Medicare claims from 1998 through 2004 for colonoscopy, and from 2000 through 2004 for sigmoidoscopy or barium enema. We classified patients with endoscopies during these intervals as compliant with no CRC testing needed in 2005. We classified enrollees who received any Medicare-covered CRC test in 2005 as receiving a CRC test in 2005. If we found no previous CRC test, and no test in 2005, we classified enrollees as not tested in 2005 with no evidence of being current with CRC testing.

### Classification of physician specialty

We identified physician specialty by linking UPINs to the Centers for Medicare and Medicaid Services Physician Specialty table, which contains up to 3 specialties per physician. We classified physician specialties as primary care, mixed specialty, or nonprimary care. We grouped internal medicine, family medicine, general practice, preventive medicine, geriatric medicine, obstetrician/gynecologists, nurse practitioners, and physician assistants under primary care. We classified physicians with more than 1 specialty designation, 1 of which was primary care (for example, internal medicine and cardiology) as mixed specialty. We classified physicians with only nonprimary care specialties as nonprimary care. We limited analysis to visits with medical doctors, doctors of osteopathy, nurse practitioners, physician assistants, certified clinical nurse specialists, and family nurse practitioners, which included 97% of all visits in the applicable study windows.

### Calculating physician visits

We were interested in the physician visits during the year before CRC testing. For enrollees with a CRC test in 2005, we examined the 12 months before the date of the CRC test; for noncompliant enrollees who had no CRC test, we used claims for the calendar year 2004 to calculate the frequency of physician visits. We used the term "physician visits" for both doctor and nurse practitioner or physician assistant visits. We excluded visits for critical care and visits to physicians with specialties not involving patient contact (eg, radiology). We excluded visits with physicians whose specialty could not be determined, such as those with bad UPINs or out-of-state physicians (3.5% of visits). Only 1 visit per day was counted for each physician–patient encounter.

We classified enrollees on the basis of specialties of the physicians they visited according to the following 7 categories: 1) only primary care physicians; 2) only nonprimary care physicians; 3) physicians with mixed specialties (1 of which was a primary care specialty); 4) both primary care and mixed-specialty physicians; 5) both primary care and nonprimary care physicians; 6) mixed-specialty and nonprimary care physicians; and 7) primary care, nonprimary care, and mixed-specialty physicians.

### Statistical analyses

We present demographic and enrollment characteristics for the 3 groups of enrollees: those tested in 2005 (any test in 2005), those eligible to be tested but not receiving a test in 2005 (not compliant and not tested in 2005), and those who did not need a CRC test (compliant — no test needed). We analyzed physician visits for 2 groups: those who were and were not tested in 2005. Because the data used for these analyses are population-based, we used descriptive rather than inferential statistics.

We used multivariate logistic regression to examine the relative importance of the number and type of physician visits on receipt of a CRC test in 2005. As measures of association between frequency and type of physician visits and receipt of a CRC test, we calculated adjusted odds ratios (ORs) with 95% confidence intervals (95% CIs). We present 3 models: Model 1, among all enrollees, in which we examine the association between number of visits and receipt of CRC testing; Model 2, conducted only for enrollees who had 1 or more physician visits, in which we examine number of visits and physician specialty; and Model 3, among enrollees who had a primary care visit, in which we examine the relationship of number of visits and the type of primary care provider seen. We adjusted all models for enrollee age, race/ethnicity, sex, original reason for eligibility (age or disabling condition), entitlement status (state buy-in or no state buy-in), state of residence, and number of comorbid conditions. Entitlement status refers to eligibility for state Medicaid programs to pay Medicare premiums and serves as a marker for low income.

## Results

The study included 1,108,424 North Carolina and South Carolina Medicare enrollees aged 65 to 85 (Table 1). Of these, 46.5% were not compliant and not tested in 2005; 21.9% had a CRC test in 2005; and 31.6% were compliant with CRC tests and were excluded from further analyses. Of those who needed a CRC test in 2005 (n = 757,594), 68% did not receive one. Compared with enrollees who had any CRC test in 2005, those who did not receive a test were more likely to be of minority race or Hispanic ethnicity (20.4% vs 14.4%); to have originally entered Medicare because of a disabling condition (11.1% vs 8.8%); and to be eligible for a state buy-in program (18.3% vs 10.3%). Those with no test in 2005 were more likely to be classified as unknown in terms of comorbid conditions (15.6% vs 4.4%), likely because of a limited number of health claims in 2004.

Lack of a physician visit during the previous year was higher among enrollees with no CRC tests compared with enrollees who had a CRC test in 2005 (19.8% vs 5.4%) (Table 2). Those not tested in 2005 had, on average, fewer physician visits than those who were tested (4.7 visits vs 7 visits). Among enrollees who were not tested in 2005, 45% received care exclusively from primary care physicians (Table 3). Enrollees without a test in 2005 were less likely than enrollees with a test to have received care from both primary care and nonprimary care physicians (17% vs 24%) and from physicians with primary care, nonprimary care, and mixed specialties (10% vs 18%).

**Figure 1:**
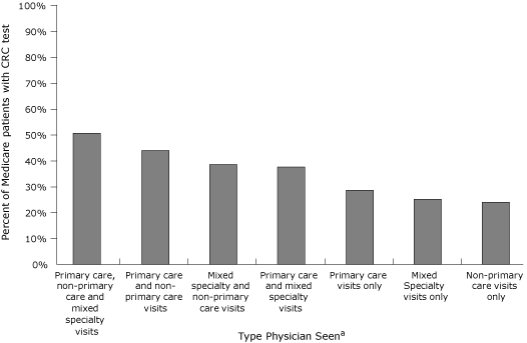
Percentage of fee-for-service Medicare enrollees current with colorectal cancer (CRC) testing, by physician type, seen by any physician, North Carolina and South Carolina, 2005.

Of all Medicare patients who received care from at least 3 different types of physicians (primary care, nonprimary care, and mixed-specialty physicians), only half had a CRC test in 2005 (Figure 1). Less than one-third of patients who had only primary care, nonprimary care, or mixed-specialty visits had a CRC test.

**Table:** 

**Type of Physician Seen^a^**	**Medicare Patients With Colorectal Cancer Test, % **
Primary care, nonprimary care, and mixed specialty visits	50.6
Primary care and nonprimary care visits	43.9
Mixed specialty and nonprimary care visits	38.5
Primary care and mixed specialty visits	37.5
Primary care visits only	28.7
Mixed-specialty visits only	25.0
Nonprimary care visits only	23.9

a Physician categories: 1) primary care: internal medicine, family medicine, general practice, preventive medicine, geriatric medicine, obstetrician/gynecologists, nurse practitioners, and physician assistants; 2) mixed specialty: physicians with more than 1 specialty listed, 1 of which was a primary care specialty; 3) nonprimary care: physicians with only nonprimary care specialties.

**Figure 2:**
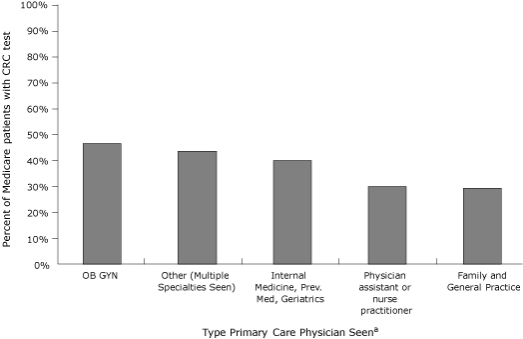
Percentage of fee-for-service North Carolina and South Carolina Medicare enrollees who were seen by primary care physicians in 2005, current with CRC testing, by primary care specialty

Medicare patients with obstetrician/gynecologist visits were most likely to be tested in 2005 (47%), followed by patients who had seen physicians with multiple specialties (Figure 2). Patients who had visits with family or general practice physicians were least likely to be tested (29%). No primary care specialty was without missed opportunities: less than half of the patients with primary care visits in 2005 had a CRC test, regardless of the primary care specialty of the physicians.

**Table:** 

**Type of Primary Physician Seen**	**Medicare Patients With Colorectal Cancer Test, % **
Obstetrics and gynecology	46.6
Other (multiple specialties seen)	43.5
Internal medicine, preventive medicine, geriatrics	40.0
Physician assistant or nurse practitioner	29.9
Family and general practice	29.1

Abbreviations: CRC, colorectal cancer; OB-GYN, obstetrics and gynecology; Prev Med, preventive medicine.

Model 1 (Table 4) revealed a strong, positive association between the number of visits and having a test, with a monotonic increase in the likelihood of having a CRC test as the number of visits increased. Enrollees with 1 to 5 visits were more than twice as likely to have had a test as those with no visits (OR, 2.6; 95% CI, 2.6-2.7). Enrollees with 21 or more visits were 8 times as likely to have had a test as those with no visits (OR, 8.3; 95% CI, 7.9-8.6).

Model 2 (Table 4) showed that enrollees whose visits included contact with a primary care physician were significantly more likely to be tested in 2005 than enrollees with nonprimary care visits. Odds ratios for all categories of physician visits that included primary care visits were significantly greater than 1. Although the number of visits remained an important predictor, the effect was attenuated by type of physician included in the model.

In Model 3 (Table 4), enrollees who saw family or general medicine physicians were less likely to have had a CRC test (OR, 0.61; 95% CI, 0.60-0.62) than those who visited physicians with internal medicine, preventive medicine, or geriatric specialties. Enrollees who saw obstetrician/gynecologists were more likely to have had a CRC test (OR, 1.23; 95% CI, 1.17-1.29).

In all 3 models, demographic characteristics were significantly associated with receipt of CRC test. Medicare enrollees who were older, minority race or Hispanic ethnicity, or male were less likely to be tested. Enrollees originally eligible because of disability and those entitled to state buy-in (a marker for low income) were also less likely to have had a CRC test. Enrollees with evidence of any comorbid conditions were less likely to have had a CRC test. State variation was also observed: enrollees from South Carolina were more likely to have had CRC tests than those from North Carolina.

## Discussion

Almost half (47%) of the Medicare population in 2 Southern states was in need of, and did not receive, a CRC test in 2005, a finding consistent with earlier studies showing low CRC test use rates despite Medicare coverage (14-16). We found that having any physician office visit, increased number of office visits, and visits with primary care physicians were associated with increased CRC test use. We also found that a number of enrollees did not regularly see physicians, and many of those who did have physician contact remained untested.

We identified missed opportunities by all provider types to promote CRC testing. For most specialties, most of their Medicare patients were in need of a CRC test at the time of the visit and did not receive one. The characteristics of the untested group reveal a potentially vulnerable population — those with Medicaid eligibility, who may have disabling conditions and multiple chronic conditions. Most are accessing medical care, but their access to preventive services remains "unrealized." Our findings highlight the gap between coverage and receipt of CRC test and demonstrate the influence of physician visits. We identify 3 categories of Medicare enrollees who are untested: those with no visits, those with primary care visits, and those with visits to multiple types of physicians. This categorization suggests that different strategies may be needed to improve CRC testing in each group.

Previous research has shown preventive or periodic health exams are associated with CRC test use (17). Yet, we found almost 20% of enrollees who needed a CRC test had no physician contact during the year of observation. In 2011, Medicare will allow 1 annual wellness visit during which counseling on CRC screening could occur. However, prior experience indicates that coverage of a wellness visit by itself will not assure Medicare enrollees use it — in 2006 and 2007, only 3% of enrollees used the "Welcome to Medicare" visit (18). Outreach to new Medicare enrollees, and "in-reach" to recall patients who have not recently been seen, may improve CRC testing rates (19).

As a stand-alone strategy, encouraging enrollees to visit primary care physicians may not result in substantial improvements in CRC testing at the population level (20). In our cohort, many enrollees remained untested regardless of the frequency of visits. Our findings are consistent with earlier studies reporting that contact with physicians is not sufficient to ensure that CRC testing will be completed (5,15,21,22). Greater understanding of tools and systems is needed to support physicians in the promotion of CRC testing. Factors identified as barriers to physicians offering CRC testing include the limited amount of time for a patient visit, competing priorities, and lack of office systems to facilitate screening (23-25). Increased CRC screening referrals have been documented through the use of simple tools such as chart stickers and reminders (26). A toolbox containing sample policies, reminder systems, and communication approaches is available from the National Colorectal Cancer Roundtable to assist physicians in increasing CRC testing (www.nccrt.org).

Physician attitudes toward cancer screening may vary by specialty. The higher rate of CRC testing we found among enrollees with an obstetrician/gynecologist visit is consistent with findings from an earlier study showing significantly higher rates of mammography referral for patients treated by obstetrician/gynecologists compared with other primary care physicians (27).

Although strategies to improve CRC testing typically target primary care physicians (28,29), we found that a combination of primary care and specialist visits was associated with increased CRC testing. A recent study reported that specialists spend a substantial portion of their time providing routine care and preventive services to their patients (30). Electronic health records (EHRs) offer potential for improving CRC testing among all physicians, including specialists, by alerting physicians to patients' test status. EHRs could identify and recall patients for screening, remind office staff to counsel patients about screening, and monitor screening compliance. An EHR system with the ability to exchange information across settings may be the only feasible way to manage the multiplicity of communication that needs to occur between the many physicians providing care to Medicare enrollees.

### Study limitations and strengths

CRC testing rates are known to vary by geographic region (3,31,32). Different patterns may be observed in other locations. Current US Preventive Services Task Force recommendations on CRC screening recommend against routine screening of people aged 76 to 85 (33). We included this age group in this study because we were targeting unscreened Medicare enrollees. CRC testing for some enrollees in this age group may not be appropriate.

The strong associations observed between number and type of physician visits and receipt of CRC testing are open to several interpretations. They may represent causal relationships, meaning increasing the number and type of physician visits would raise CRC test rates. However, it is also possible the associations are driven by medical conditions that cause patients to visit doctors more frequently or other characteristics that may independently lead to being tested for CRC.

Medicare data provide the opportunity to study a large population, examining care received from multiple providers to accurately measure CRC test status (12,13). They are not, however, without limitations. Tests conducted before patients enter Medicare are not available. Younger enrollees who had endoscopy before entering Medicare may not be due for another test. Additionally, we had access to claims from an 8-year window (1998-2005). Medicare enrollees who had colonoscopy during 1996 through 1997 would be incorrectly classified in our data as untested. Medicare did not cover screening for CRC during that time period, and diagnostic test use was low. The effect of these data limitations is minimal, as our population-based rates compare favorably with rates from national surveys (3,34).

### Conclusions

Increased CRC screening would reduce CRC death, but much work remains to be done to realize that benefit. Working with the Medicare population to increase "realized" access for those with insurance may inform approaches to access as we move toward health insurance reform.

## Figures and Tables

**Table 1:** Characteristics of Fee-For-Service Medicare Enrollees, by Colorectal Cancer Test Status, North Carolina and South Carolina, 2005

Characteristic	Not Compliant and Not Tested^a^ (n = 515,193), No. (%)	Any Test^b^ (n = 242,401), No. (%)	Compliant, No Test Needed^c^ (n = 350,830), No. (%)
**Age, y**			
65-74	299,204 (58.1)	148,662 (61.3)	191,528 (54.6)
75-85	215,989 (41.9)	93,739 (38.7)	159,302 (45.4)
**Race/ethnicity**			
White	409,936 (79.6)	207,393 (85.6)	296,148 (84.4)
African American	97,314 (18.9)	32,625 (13.5)	51,079 (14.6)
Asian	2,441 (0.5)	565 (0.2)	857 (0.2)
Hispanic	1,092 (0.2)	257 (0.1)	416 (0.1)
Native American	1,433 (0.3)	472 (0.2)	860 (0.2)
Other	2,977 (0.6)	1,089 (0.4)	1,470 (0.4)
**Sex**			
Female	294,946 (57.2)	147,951 (61.0)	204,189 (58.2)
**Eligibility reason**			
Aged	457,833 (88.9)	221,121 (91.2)	312,841 (89.2)
Disabled	57,360 (11.1)	21,280 (8.8)	37,989 (10.8)
**Entitlement^d^**			
Medicare only	420,663 (81.7)	217,495 (89.7)	303,578 (86.5)
State buy-in	94,530 (18.3)	24,906 (10.3)	47,252 (13.5)
**State**			
North Carolina	337,154 (65.4)	153,390 (63.3)	231,630 (66.0)
South Carolina	178,039 (34.6)	89,011 (36.7)	119,200 (34.0)
**Comorbid conditions^e^**			
None	310,600 (60.3)	167,894 (69.3)	229,968 (65.5)
1	91,557 (17.8)	47,956 (19.8)	75,889 (21.6)
≥2	32,461 (6.3)	15,963 (6.6)	30,654 (8.7)
Unknown	80,575 (15.6)	10,588 (4.4)	14,319 (4.1)

Abbreviation: FOBT, fecal occult blood test.

a No claims-based evidence of a colonoscopy during 1998-2005, or a sigmoidoscopy or barium enema during 2000-2005, and no FOBT claim in 2005.

b A claim for colonoscopy, sigmoidoscopy, barium enema, or FOBT in 2005.

c Claims-based evidence of a colonoscopy during 1998-2004, or a sigmoidoscopy or barium enema during 2000-2004.

d State buy-in indicates the enrollee is eligible for Medicaid to "buy" Medicare coverage.

e Classified using Charlson index (11) modified for use with claims data. Categories indicate the number of Charlson conditions identified. Unknown indicates insufficient claims available to assess comorbidity.

**Table 2:** Physician Office Visits by Fee-for-Service Medicare Enrollees, by Colorectal Cancer Test Status, North Carolina and South Carolina, 2005

No. of Physician Visits^a^	Not Compliant and Not Tested^b^ (n = 515,193), No. (%)	Any Test^c^ (n = 242,401), No. (%)
0	101,879 (19.8)	13,025 (5.4)
1-5	244,450 (47.3)	106,804 (44.1)
6-10	112,919 (21.9)	73,174 (30.2)
11-15	36,219 (7.0)	29,369 (12.1)
16-20	12,161 (2.4)	11,668 (4.8)
≥21	7,565 (1.5)	8,361 (3.4)
Mean no. of visits	4.7	7.0

Abbreviation: FOBT, fecal occult blood test.

a Visits to physicians with surgical, critical care, or other nonpatient-contact specialty are not included. The time window for counting visits varies by study group: for those not compliant and not tested in 2005, calendar year 2004 claims were used; for those tested in 2005, a 12-month window ending with the month before their first test in 2005 was used.

b No claims-based evidence of a colonoscopy during 1998-2005, or a sigmoidoscopy or barium enema during 2000-2005, and no FOBT claim in 2005.

c A claim for colonoscopy, sigmoidoscopy, barium enema, or FOBT in 2005.

**Table 3:** Type of Physicians Seen by Fee-for-Service Medicare Enrollees With Any Visit, North Carolina and South Carolina, 2005

Type of Physician(s) Seen		
Enrollees with any physician visit	Not Compliant and Not Tested^a^ No. (%) (n = 413,314)	Any Test^b^ No. (%) (n = 229,376)
Nonprimary care physicians only	13,641 (3.3)	4,285 (1.9)
Primary care physicians only	188,052 (45.5)	75,514 (32.9)
Physicians with mixed specialty only	29,488 (7.1)	9,829 (4.3)
Primary care and mixed-specialty physicians	60,358 (14.6)	36,282 (15.8)
Primary care and nonprimary care physicians	68,930 (16.7)	54,038 (23.6)
Physicians with mixed specialty and nonprimary care physicians	11,887 (2.9)	7,449 (3.2)
Primary care, nonprimary care, and mixed-specialty physicians	40,958 (9.9)	41,979 (18.3)

**Enrollees with a primary care visit**	**(n = 358,298)**	**(n = 207,813)**

Internal medicine, preventive medicine, geriatrics	103,851 (29.0)	69,289 (33.3)
Family and general practice	149,925 (41.8)	61,643 (29.7)
Obstetrician/gynecologist	3,603 (1.0)	3,142 (1.5)
Physician assistant or nurse practitioner	11,410 (3.2)	4,862 (2.3)
Other (multiple specialties)	89,509 (25.0)	68,877 (33.1)

Abbreviation: FOBT, fecal occult blood test.

a No claims-based evidence of a colonoscopy during 1998-2005, or a sigmoidoscopy or barium enema during 2000-2005, and no FOBT claim in 2005.

b A claim for colonoscopy, sigmoidoscopy, barium enema, or FOBT in 2005.

**Table 4:** Influence of Number and Type of Physician Office Visits on Receipt of Colorectal Cancer Testing by Fee for Service Medicare Enrollees, North Carolina and South Carolina, 2005

Enrollee Characteristic	Model 1: All Enrollees (n = 757,594), OR (95% CI)^a^	Model 2: Enrollees With Any Physician Visit (n = 642,690), OR (95% CI)^b^	Model 3: Enrollees With a Visit to a Primary Care Physician (n = 566,111), OR (95% CI)^c^
**Age, y **			
65-69	1.0 [Referent]		
70-74	1.18 (1.17-1.20)	1.19 (1.17-1.21)	1.20 (1.18-1.22)
75-79	1.02 (1.01-1.03)	1.02 (1.01-1.04)	1.02 (1.00-1.03)
80-85	0.70 (0.69-0.71)	0.70 (0.69-0.72)	0.69 (0.68-0.70)
**Race/ethnicity**			
White	1.0 [Referent]		
African American	0.83 (0.82-0.84)	0.90 (0.88-0.91)	0.85 (0.83-0.86)
Asian	0.69 (0.62-0.75)	0.69 (0.63-0.77)	0.66 (0.59-0.74)
Hispanic	0.72 (0.63-0.83)	0.71 (0.61-0.82)	0.71 (0.61-0.84)
Native American	0.80 (0.72-0.89)	0.83 (0.75-0.93)	0.78 (0.69-0.88)
Other Race	0.85 (0.79-0.91)	0.88 (0.81-0.95)	0.83 (0.77-0.91)
**Sex **			
Female	1.0 [Referent]		
Male	0.90 (0.89-0.91)	0.91 (0.90-0.92)	0.98 (0.97-0.99)
**Entitlement^d^ **			
No state buy-in	1.0 [Referent]	1.0 [Referent]	1.0 [Referent]
State buy-in	0.54 (0.53-0.55)	0.55 (0.54-0.56)	0.55 (0.54-0.56)
**Eligibility reason **			
Age	1.0 [Referent]		
Disability	0.82 (0.80-0.83)	0.82 (0.80-0.83)	0.82 (0.81-0.84)
**State of residence**			
North Carolina	1.0 [Referent]		
South Carolina	1.12 (1.10-1.13)	1.09 (1.08-1.10)	1.15 (1.14-1.17)
**Comorbid conditions^e^ **			
0	1.0 [Referent]		
1	0.82 (0.81-0.83)	0.80 (0.79-0.82)	0.81 (0.79-0.82)
≥2	0.69 (0.68-0.71)	0.65 (0.64-0.67)	0.66 (0.65-0.68)
Unknown	0.65 (0.63-0.67)	2.26 (2.17-2.35)	1.95 (1.87-2.04)
**Number of visits**			
0	1.0 [Referent]	NA	NA
1-5	2.62 (2.55-2.69)	NA	NA
6-10	4.19 (4.07-4.31)	NA	NA
11-15	5.65 (5.48-5.83)	NA	NA
16-20	6.91 (6.66-7.18)	NA	NA
≥21	8.27 (7.93-8.63)	NA	NA
**Number of visits * ***			
1-5	NA	1.0 [Referent]	
6-10	NA	1.29 (1.27-1.31)	1.53 (1.51-1.55)
11-15	NA	1.56 (1.53-1.59)	2.02 (1.99-2.06)
16-20	NA	1.81 (1.76-1.87)	2.44 (2.37-2.51)
≥21	NA	2.07 (2.00-2.15)	2.87 (2.77-2.97)
**Type of physicians seen^f^ **			
Nonprimary care only	NA	1.0 [Referent]	NA
Primary care only	NA	1.26 (1.22-1.31)	NA
Mixed specialties only	NA	1.08 (1.03-1.12)	NA
Primary care and mixed specialty	NA	1.81 (1.74-1.88)	NA
Primary care and nonprimary care	NA	2.21 (2.13-2.30)	NA
Mixed specialty and nonprimary care	NA	1.84 (1.76-1.93)	NA
Primary care, nonprimary care and mixed specialty	NA	2.66 (2.56-3.77)	NA
**Type of primary care physician* ***			
Internal medicine, preventive medicine, or geriatrics only	NA	NA	1.0 [Referent]
Family and general practice only	NA	NA	0.61 (0.60-0.62)
Obstetrician/gynecologist only	NA	NA	1.23 (1.17-1.29)
Physician assistant or nurse practitioner only	NA	NA	0.67 (0.64-0.69)
Other (multiple specialties)	NA	NA	1.02 (1.00-1.03)

Abbreviations: OR, odds ratio; CI, confidence interval; NA, not applicable because variable not included in the model.

a For Model 1, the association between number of visits and receipt of colorectal cancer testing was examined among all enrollees.

b For Model 2, the association between number of visits and physician specialty were examined only for enrollees who had 1 or more physician visits.

c For Model 3, the association between number of visits and the type of primary care provider seen was examined for enrollees who had primary care visits.

d State buy-in indicates the enrollee is eligible for Medicaid to "buy" Medicare coverage.

e Classified using Charlson index (11) modified for use with claims data. Categories indicate the number of Charlson conditions identified. Unknown indicates insufficient claims available to assess comorbidity.

f Physician categories: 1) primary care: internal medicine, family medicine, general practice, preventive medicine, geriatric medicine, obstetrians/gynecologists, nurse practitioners and physician assistants; 2) mixed specialty: physicians with more than 1 specialty listed, 1 of which was a primary care specialty; 3) nonprimary care: physicians with only nonprimary care specialties.

## Appendix A. ICD-9 Codes Used to Identify and Exclude People Who Had Above Average Risk for Colorectal Cancer

**Table:**

Clinical condition	ICD-9 Code
Malignant neoplasm of other and ill-defined sites within the digestive organs and peritoneum	159
Carcinoma in situ of colon	230.3
Carcinoma in situ of rectum	230.4
Regional enteritis	555
Ulcerative colitis	556
Diverticulitis	562.01, 562.03, 562.11, 562.13

Abbreviation: ICD-9, *International Classification of Diseases, 9th Revision, Clinical Modification*.

## Appendix B. Procedure codes for identifying colorectal cancer tests

**Table:**

Type of Test	ICD-9 Codes	HCPCS Codes	CPT Codes
Fecal occult blood test (FOBT)	NA	G0107	82270, 82274

Sigmoidoscopy	45.24 45.42 48.21-48.25	G0104	45300 45303 45305 45307–45309 45327 45330 45331 45332 45333 45334 45337–45339 45340 45341 45342 45345

Colonoscopy	45.21-45.23 45.25	G0105 (high risk) G0121 (nonhigh risk)	45355 45378 45379 45380 45381 45382–45385 45386 45387 44388–44394 44397

Double contrast barium enema	NA	G0106 G0120 G0122	74270 74280

Abbreviation: ICD-9, *International Classification of Diseases, 9th Revision, Clinical Modification*; HCPCS, Healthcare Common Procedure Coding System; CPT, Current Procedural Terminology.
